# Mesenchymal Stem Cells in Combination with Hyaluronic Acid for Articular Cartilage Defects

**DOI:** 10.1038/s41598-018-27737-y

**Published:** 2018-07-02

**Authors:** Lang Li, Xin Duan, Zhaoxin Fan, Long Chen, Fei Xing, Zhao Xu, Qiang Chen, Zhou Xiang

**Affiliations:** 1 Department of Orthopedics, West China Hospital, Sichuan University, Chengdu, Sichuan 610016 China; 2 Research Center for Stem Cell and Regenerative Medicine, Sichuan Neo-life Stem Cell Biotech INC, Jinquanlu15#, Chengdu, Sichuan 610016 China; 3 Department of Orthopedics, Guizhou Provincial People’s Hospital, Guiyang, Guizhou 550002 China; 4 Department of Anesthesia, West China Hospital, Sichuan University, Chengdu, Sichuan 610016 China; 5 Center for Stem Cell Research & Application, Institute of Blood Transfusion, Chinese Academy of Medical Sciences and Peking Union Medical College, Chengdu, Sichuan 610016 China

## Abstract

Mesenchymal stem cells (MSCs) and hyaluronic acid (HA) have been found in previous studies to have great potential for medical use. This study aimed to investigate the therapeutic effects of bone marrow mesenchymal stem cells (BMSCs) combined with HA on articular cartilage repair in canines. Twenty-four healthy canines (48 knee-joints), male or female with weight ranging from 5 to 6 kg, were operated on to induce cartilage defect model and divided into 3 groups randomly which received different treatments: BMSCs plus HA (BMSCs-HA), HA alone, and saline. Twenty-eight weeks after treatment, all canines were sacrificed and analyzed by gross appearance, magnetic resonance imaging (MRI), hematoxylin-eosin (HE) staining, Masson staining, toluidine blue staining, type II collagen immunohistochemistry, gross grading scale and histological scores. MSCs plus HA regenerated more cartilage-like tissue than did HA alone or saline. According to the macroscopic evaluation and histological assessment score, treatment with MSCs plus HA also lead to significant improvement in cartilage defects compared to those in the other 2 treatment groups (*P* < 0.05). These findings suggested that allogeneic BMSCs plus HA rather than HA alone was effective in promoting the formation of cartilage-like tissue for repairing cartilage defect in canines.

## Introduction

Articular cartilage is composed of chondrocyte and extracellular matrix and has an important role in joint movement including lubrication, shock absorption and conduction. However, trauma injury and many joint diseases, such as osteoarthritis(OA) can damage the cartilage layer^1^. Cartilage defects lead to restriction of joint activities, which results in pain and adverse effects on people’s lives, especially for knee articular cartilage patients. Damaged knee cartilage does not receive a sufficient blood supply, which limits its ability to repair itself^2^. Cartilage defect can also progress to OA at a later stage. The current treatment for cartilage defect includes physiotherapy, external medication, intra-articular injection, and intra-articular irrigation. Chondroplasty is an alternative method that can relieve pain^3^. However, these treatments have not been found to regenerate new cartilage-like tissue and cartilage defects can progress and develop into more severe cartilage damage^4,5^. Tissue engineering strategies combining cells and a scaffold are used to achieve cartilage regeneration^6^.

In recent years, cell therapy has been used to treat cartilage damage. Mesenchymal stem cells (MSCs) have been suggested as a potential cell fortreatment of OA because of their multiple differentiative capacity to produce cells via osteogenesis, adipogenesis, and chondrogenesis^7–10^. MSCs are used in many cell-based tissue engineering application and have been confirmed to be safe and feasible for treatment in human beings. In addition, MSCs have been found to improve clinical symptoms such as pain, disability, and physical function^11–13^. Autologous MSCs are proper sources of cells, but to obtain them requires that patients undergo an additional operation. Allogeneic bone marrow mesenchymal stem cells (BMSCs) have been used to treat cartilage defects and to promote neocartilage formation in some studies^14,15^. Hyaluronic acid (HA) is an important component of synovial fluid that protects joint cartilage by lubricating and absorbing shock^16^. HA maintains a constant concentration and sufficient viscosity in the knee joint. When OA occurs, the concentration of HA decreases, which aggravates damage to knee cartilage^17,18^. Additionally, HA can also promote cell migration^19,20^ and is suggested to be injected every 3 months for knee joint disease^21^. Many clinical studies also have reported that HA could relieve the pain of OA patients^22–24^.

In this study, we obtained BMSCs by performing a standard isolation and culture procedure. After inducing cartilage defects in canines, the therapeutic effects of injections of BMSCs plus HA, HA alone, and normal saline were compared by assessing gross appearance, evaluating MRI results, and performing histological and immunohistochemical analysis.

## Results

Three canines used for obtaining bone marrow were left for other studies, and the other 24 canines appeared to recover 1 week after the operation. No deaths occurred, no local infections developed, and all animals moved freely. Additionally, flow cytometry was performed and the results were shown in Figure S1. The positive rates of CD 166, CD 29, CD 90, CD 105 and CD 44 were 100%, 99.9%, 100%, 93.5% and 100%, respectively, and the negative rates of CD45 and CD 34 were 99.4%, and 99.8%, respectively. The antigenic profile conformed to cellular therapy criteria of MSCs. After induction in special culture, osteogenesis and chondrogenesis of BMSCs was shown in Figure S2. Extracellular matrix appeared light red after chondrogenesis, and calcium nodules were stained orange after osteogenesis.

### Gross appearance of the cartilage

**Figure 1:**
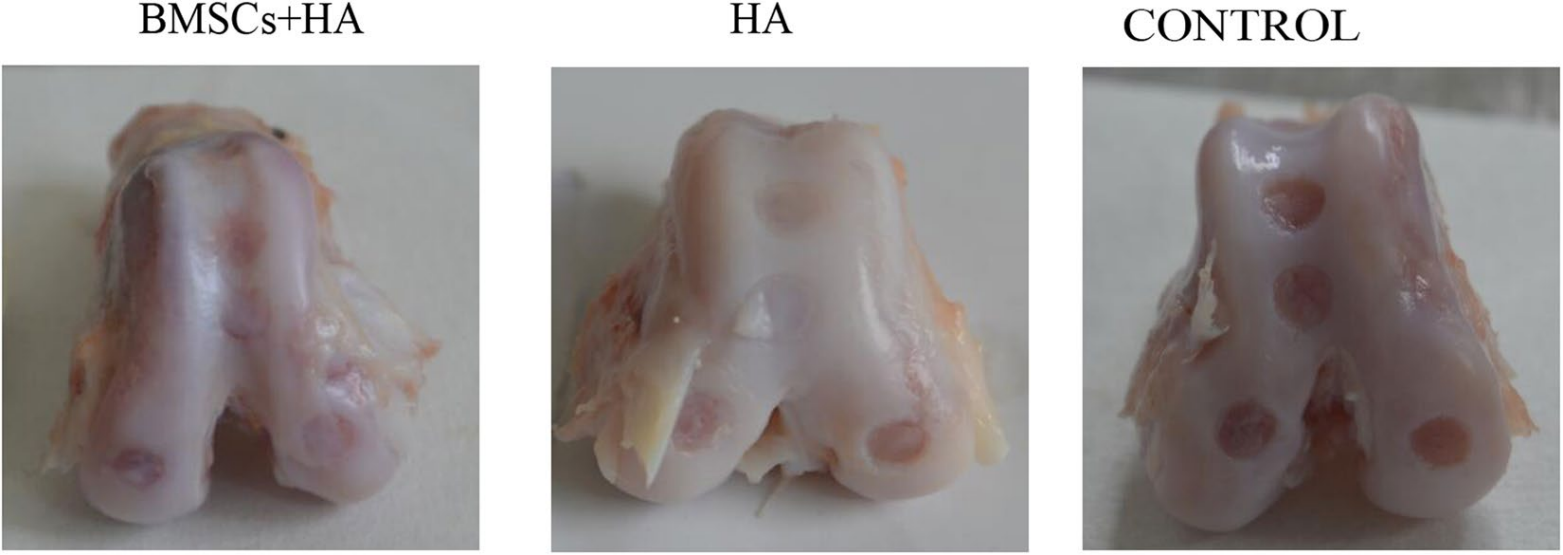
The gross appearance of the cartilage 28 weeks after injection. Representative gross appearance among the group A (BMSCs plus HA), B (HA alone) and C (control group) 28 weeks after injection.

Varying degrees of cartilage damage were sustained 28 weeks after injection. Representative gross of cartilage was shown in Fig. 1. No significant degenerative changes were observed in knee-joint cartilage except for cartilage defects (medial condyle, intercondylar groove, and lateral condyle of femur). For group A, new cartilage-like tissue was frequently observed at 4 defect sites (Fig. 1), the surface color was relatively normal, and new cartilage-like tissue connected well with surrounding cartilage tissue. For group B, new cartilage-like tissue was also frequently observed (Fig. 1), but a small scratch was visible on the junction between the defect sites and normal sites. For group C, the cartilage defects were not covered and new cartilage-like tissue was hardly observed (Fig. 1).

### Radiological analysis

**Figure 2:**
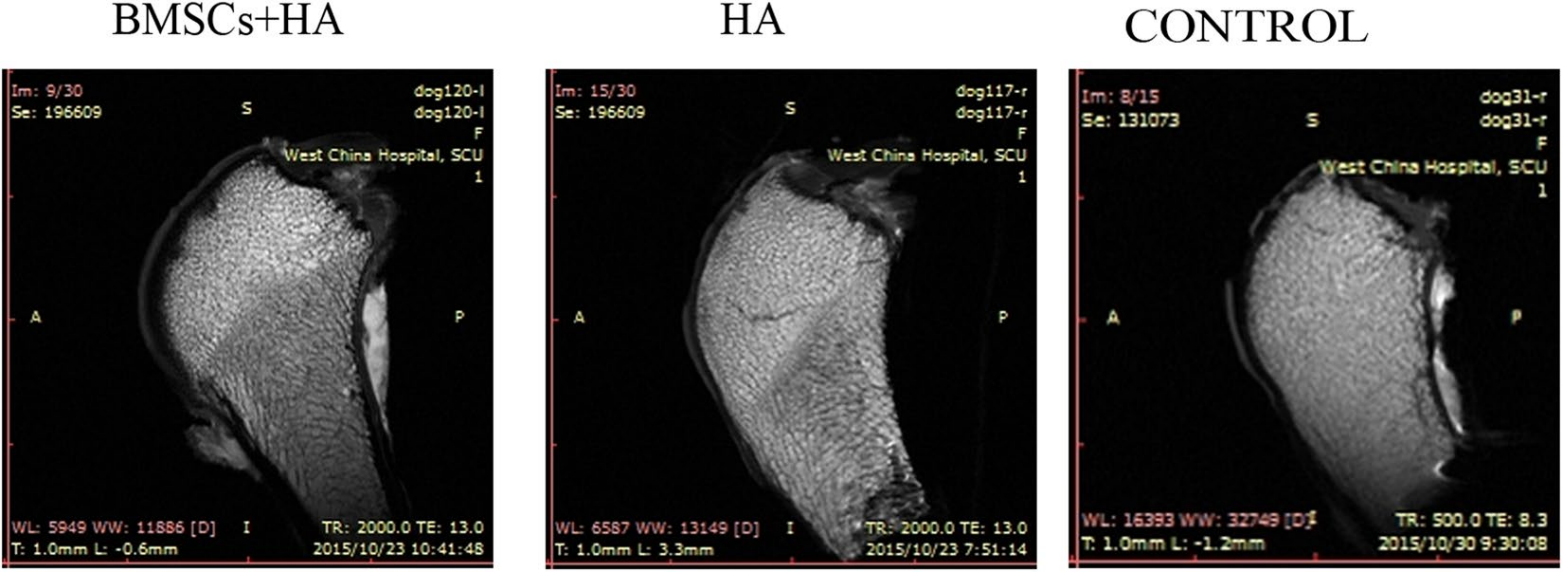
The MRI of the cartilage 28 weeks after injection. Representative MRI among the group A (BMSCs plus HA), B (HA alone) and C (control group) 28 weeks after injection.

Twenty-eight weeks after injection, MRI was performed. MRI examination of regenerated new cartilage tissue was shown in Fig. 2. For group A, cartilage-like signal was observed at defect sites, and the surface of cartilage was smooth relatively. No obvious defect was found and cartilage-like tissue with the same thickness of the surrounding normal tissue was formed (Fig. 2). For group B, cartilage-like signal was also observed, but the thicknesses of tissue at the defect sites were thinner than those at normal (Fig. 2). For group C, no cartilage-like signal was observed. These data indicated that MSCs plus HA could stimulate the formation of new cartilage-like tissue better than HA alone or normal saline for cartilage defect (Fig. 2).

### Histological and immunohistochemical analysis

**Figure 3:**
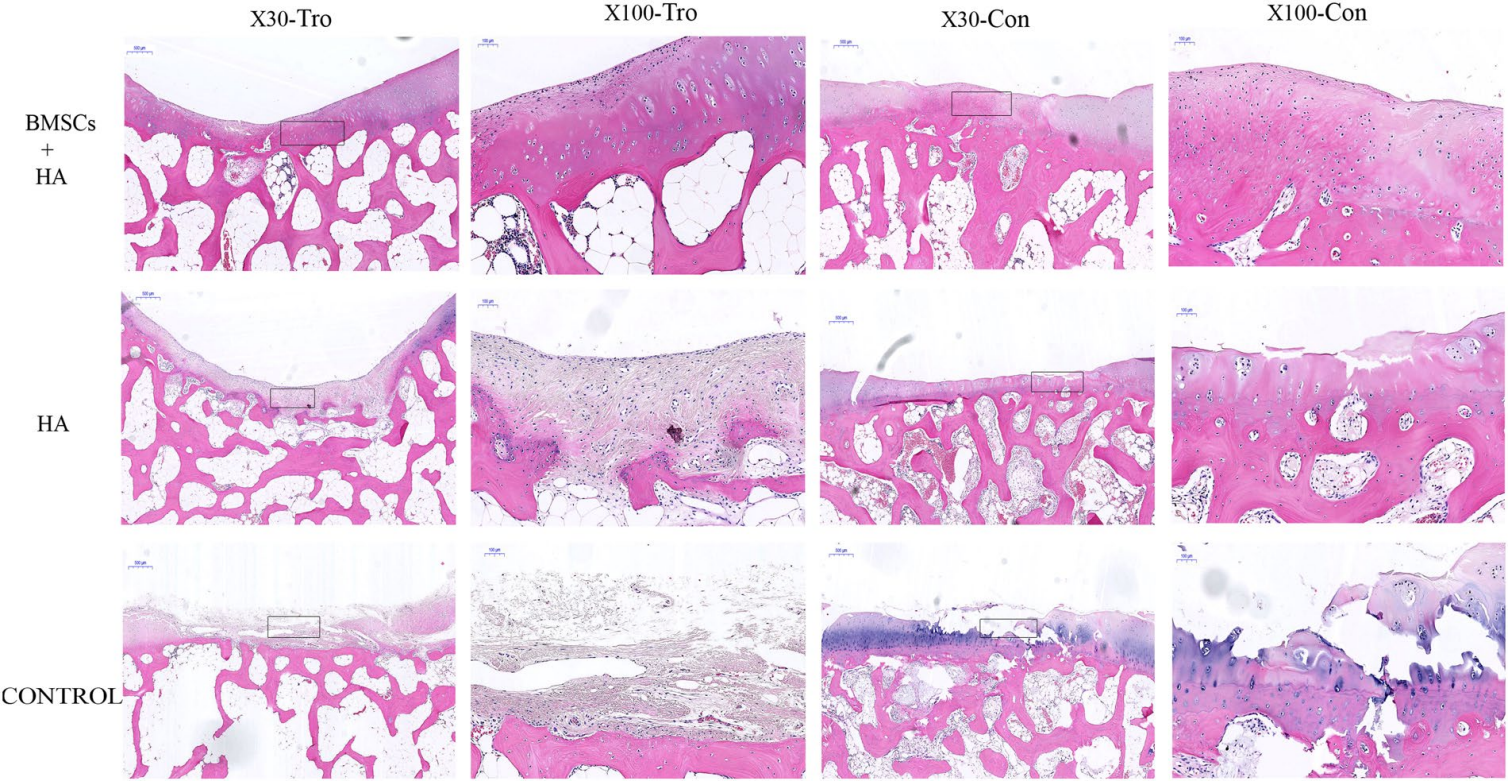
The HE staining of the cartilage 28 weeks after injection. Representative HE staining among the group A (BMSCs plus HA), B (HA alone) and C (control group) 28 weeks after injection. The black rectangle indicated repairing sites on low magnification (X30) and would be magnified to high magnification (X100). Tro: trochlear defects. Con: condyle defects. X30 (Scale bars = 500 μm), X100 (Scale bars = 100 μm).

Hematoxylin and eosin (HE) staining, Masson staining, and toluidine blue staining were performed. Representative photomicrographs of HE staining of groups A, B and C were shown in Fig. 3. In group A, tissue similar to neocartilage covered the defect site, and chondrocytes were formed and the matrix staining was normal (Fig. 3). In group B, some tissue similar to cartilage and fibrous tissue was observed (Fig. 3). However, tissue similar to neocartilage was seldom seen at the defect sites in group C (Fig. 3).

**Figure 4:**
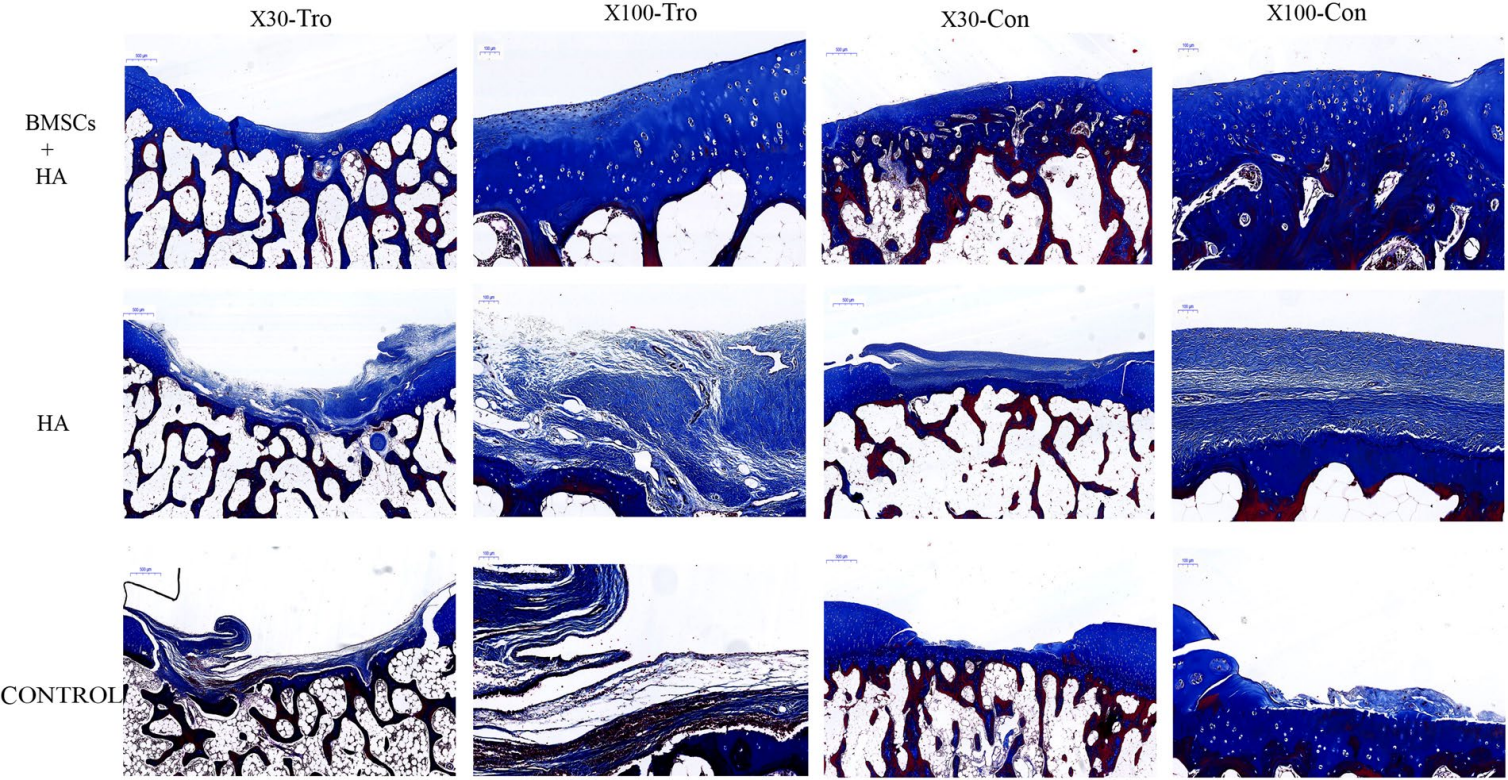
The Masson staining of the cartilage 28 weeks after injection. Representative Masson staining among the group A (BMSCs plus HA), B (HA alone) and C (control group) 28 weeks after injection. The black rectangle indicated repairing sites on low magnification (X30) and would be magnified to high magnification (X100). Tro: trochlear defects. Con: condyle defects. X30 (Scale bars = 500 μm), X100 (Scale bars = 100 μm).

Representative photomicrographs of Masson staining were shown in Fig. 4. In group A, many chondrocytes were seen, tissue similar to cartilage fiber was revealed regularly, and the color of matrix was relatively normal compared with that of normal cartilage tissue (Fig. 4). For group B, chondrocytes were hardly observed, most of the cells were non-chondrocytes, and the color of matrix was paler compared to that of normal cartilage (Fig. 4). For group C, no cartilage was observed at the defect sites (Fig. 4).

**Figure 5:**
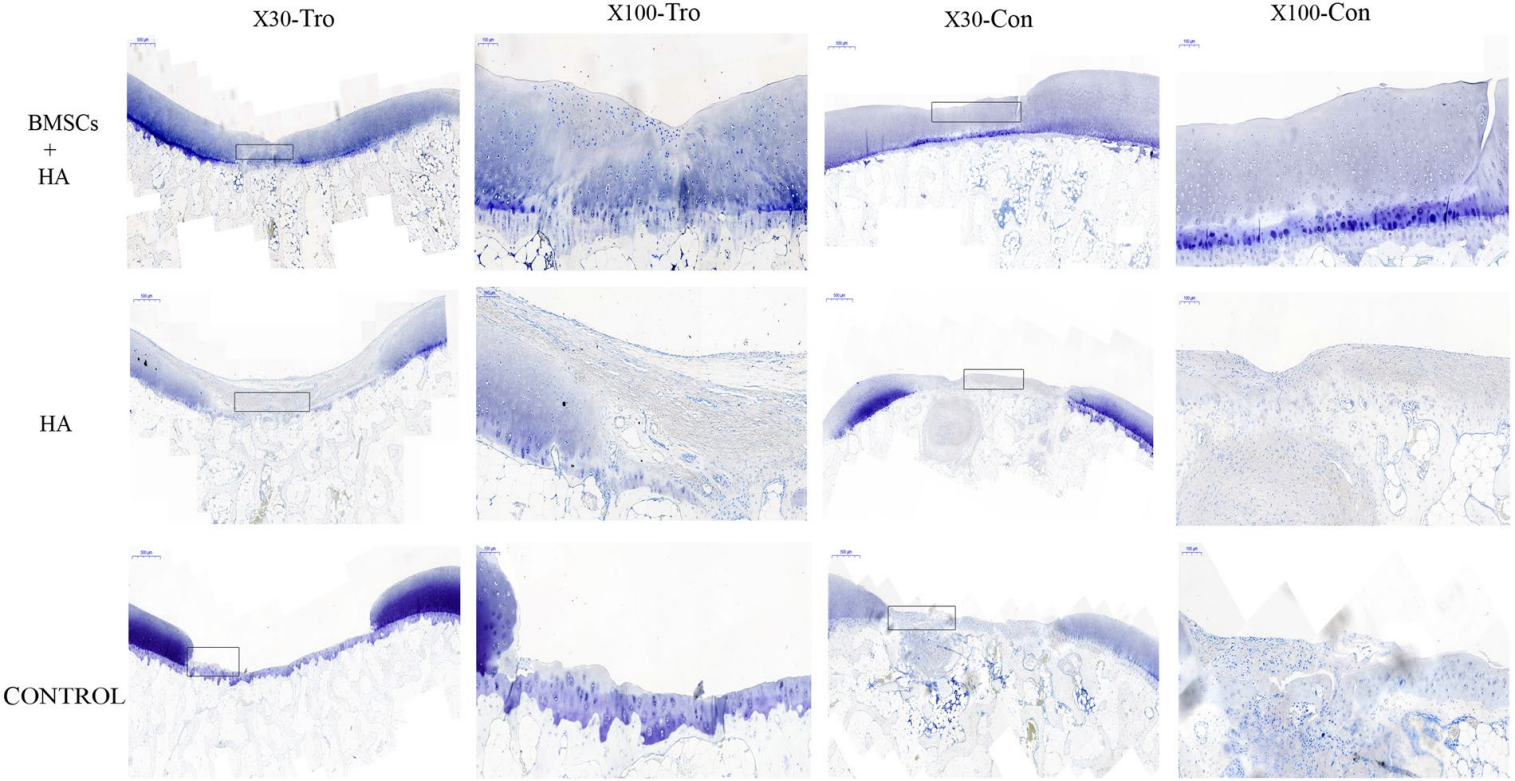
The Toluidine blue staining of the cartilage 28 weeks after injection. Representative Toluidine blue staining among the group A (BMSCs plus HA), B (HA alone) and C (control group) 28 weeks after injection. The black rectangle indicated repairing sites on low magnification (X30) and would be magnified to high magnification (X100). Tro: trochlear defects. Con: condyle defects. X30 (Scale bars = 500 μm), X100 (Scale bars = 100 μm).

Representative photomicrographs of toluidine blue staining were shown in Fig. 5. Group A showed darker blue staining at defect sites with uniform cartilage cell and clear tidemark (Fig. 5). Pale blue staining, few cartilage cells, and a mass of fibrous cell and fiber tissue were observed in group B (Fig. 5). No neocartilage tissue was observed ingroup C (Fig. 5).

**Figure 6:**
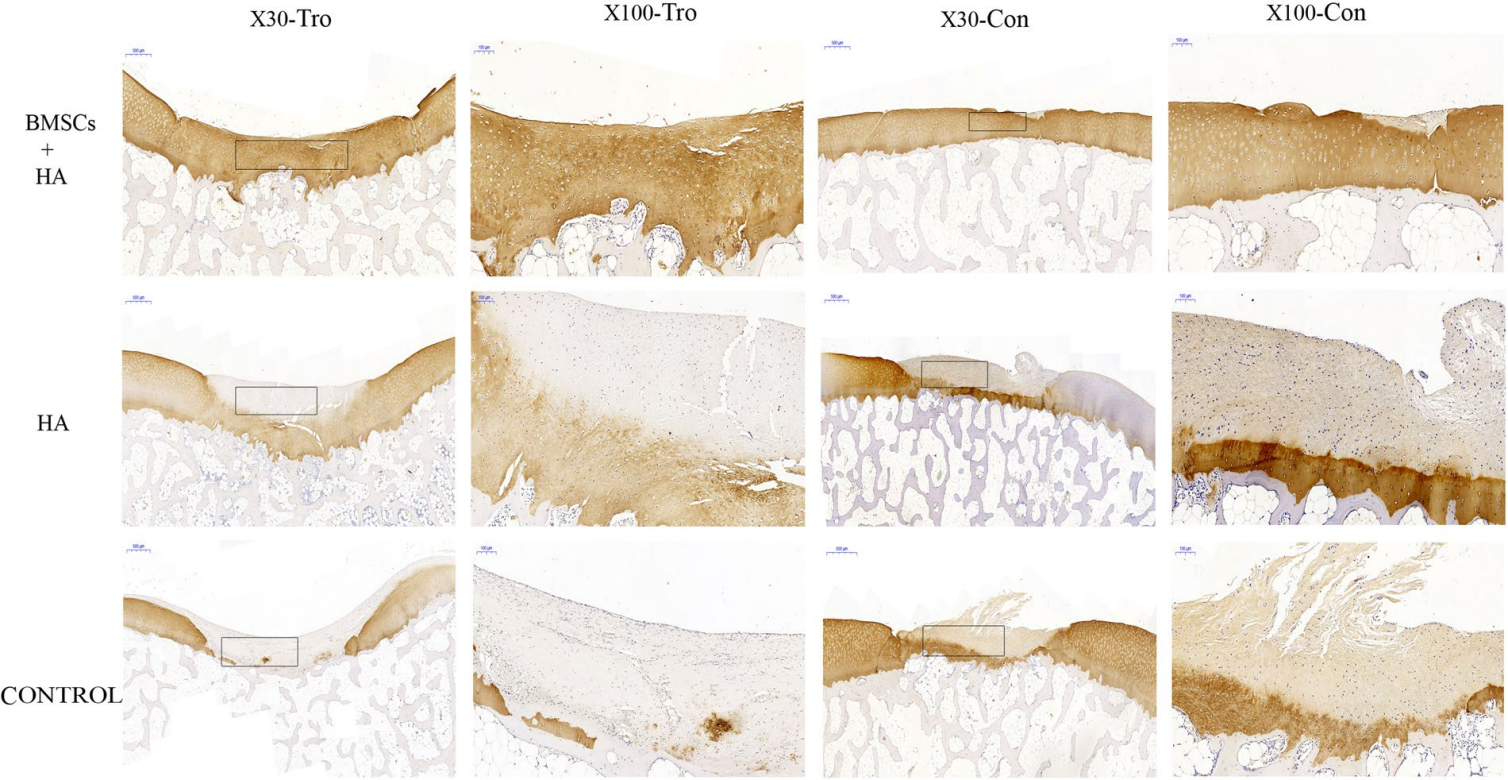
The type II collagen immunohistochemistry staining of the cartilage 28 weeks after injection. Representative type II collagen staining among the group A (BMSCs plus HA), B (HA alone) and C (control group) 28 weeks after injection. The black rectangle indicated repairing sites on low magnification (X30) and would be magnified to high magnification (X100). Tro: trochlear defects. Con: condyle defects. X30 (Scale bars = 500 μm), X100 (Scale bars = 100 μm).

Representative photomicrographs of the immunohistochemistry analysis of the neocartilage from all three groups were shown in Fig. 6. Group A exhibited large numbers of chondrocyte cells. The color of the defect sites in group A was relatively normal compared with that of the surrounding tissue, which indicated that much more type II collagen protein was formed (Fig. 6). In group B, few cells similar to chondrocytes were shown and the color was light at the defect sites (Fig. 6). In group C, no new tissue was formed (Fig. 6). These data indicated presence of more collagen fiber in group A than in groups B and C. The histochemical and immunohistochemical analysis suggested that BMSCs plus HA could stimulate the regeneration of cartilage better than HA alone.

### Gross-grading scale and histological score

**Figure 7:**
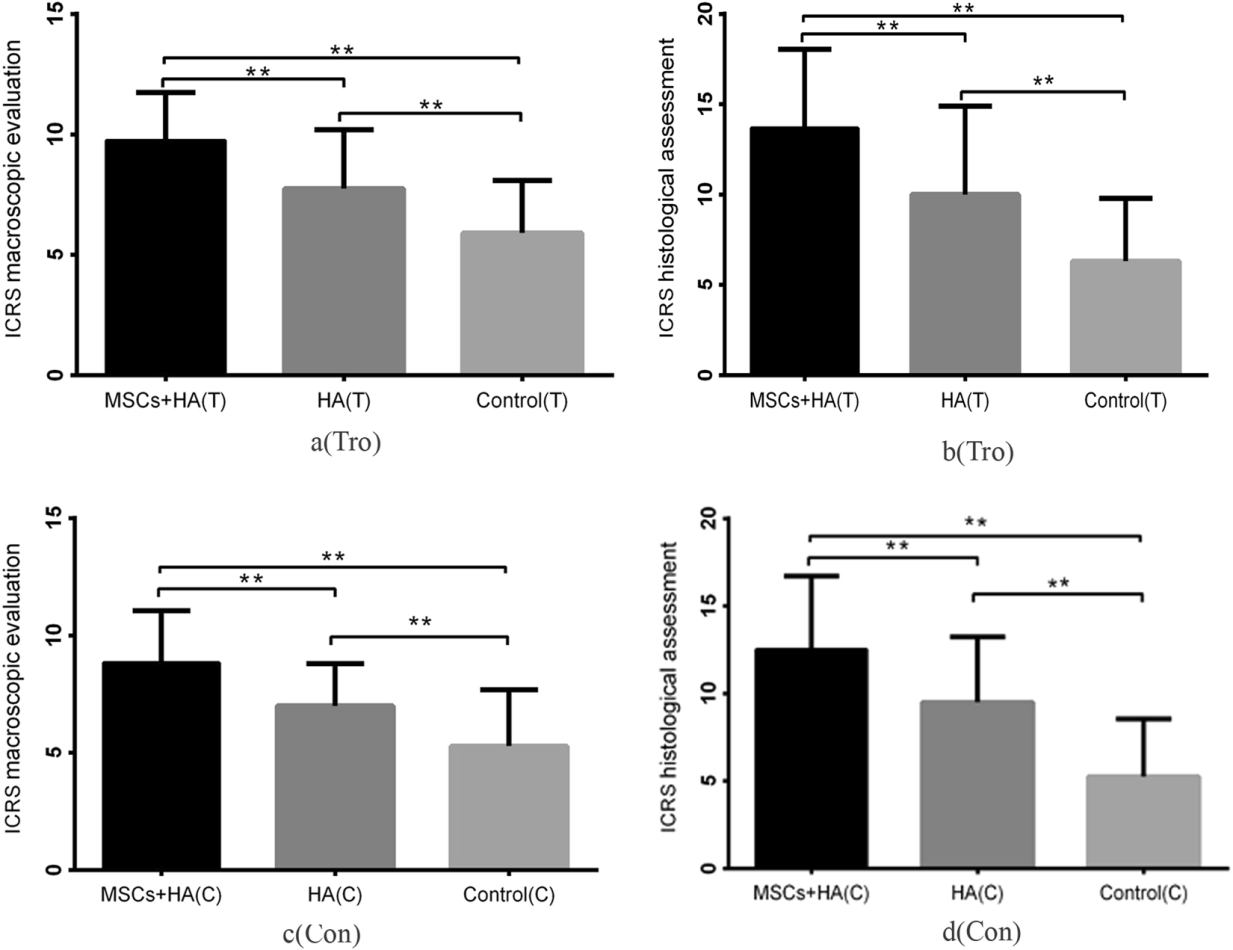
The ICRS scale for macroscopic and histological assessment 28 weeks after injection. The macroscopic and histological assessment among the group A (BMSCs plus HA), B (HA alone) and C (control group) 28 weeks after injection. Tro: trochlear defects. Con: condyle defects (*P < 0.05 **P < 0.01).

After two researchers assessed the treatment, macroscopic evaluation and histological assessment scoring were performed (Table 3). For the trochlear defects, the International Cartilage Repair Society (ICRS) macroscopic score for group A (9.75 ± 1.984) was greater than those in group B (7.75 ± 2.449) and group C (5.91 ± 2.176), with a significant difference (*P* < 0.001). The ICRS macroscopic score for group B was significantly superior to group C (*P* < 0.01) (Fig. 7a). The ICRS histological score for group A (13.75 ± 4.303) was superior to those in group B (10.19 ± 4.789, *P* < 0.001) and group C (6.44 ± 3.388, *P* < 0.001). There was also significant difference between group B and group C (*P* < 0.001) (Fig. 7b).

**Table 1:** ICRS macroscopic evaluation of cartilage repair.

Feature	score
**Degree of defect repair**	
In level with surrounding cartilage	4
75% repair of defect depth	3
50% repair of defect depth	2
25% repair of defect depth	1
0% repair of defect depth	0
**Integration to border zone**	
Complete integration with surrounding cartilage	4
Demarcating border <1 mm	3
3/4th of graft integrated, 1/4th with a notable border >1 mm width	2
1/2 of graft integrated with surrounding cartilage, 1/2 with a notable border >1 mm	1
From no contact to 1/4th of graft integrated with surrounding cartilage	0
**Macroscopic appearance**	
Intact smooth surface	4
Fibrillated surface	3
Small, scattered fissures or cracs	2
Several, small or few but large fissures	1
Total degeneration of grafted area	0
**Overall repair assessment**	
Grade I: normal	12
Grade II: nearly normal	11–8
Grade III: abnormal	7–4
Grade IV: severely abnormal	3–1

This table was adopted from ref.^54^.

**Table 2:** ICRS Visual Histological Assessment Scale.

Feature	score
**Surface**	
Smooth/continuous	3
Discontinuities/irregularities	0
**Matrix**	
Hyaline	3
Mixture: hyaline/fibrocartilage	2
Fibrocartilage	1
Fibrous tissue	0
**Cell distribution**	
Columnar	3
Mixed/columnar-clusters	2
Clusters	1
Individual cells/disorganized	0
**Cell population viability**	
Predominantly viable	3
Partially viable	1
<10% viable	0
**Subchondral Bone**	
Normal	3
Increased remodeling	2
Bone necrosis/granulation tissue	1
Detached/fracture/callus at base	0
**Cartilage mineralization (calcified cartilage)**	
Normal	3
Abnormal/inappropriate location	0

This table was adopted from ref.^55^.

**Table 3:** The basic characteristics of ICRS marcroscopic score and ICRS histological score.

gross/Histological	group	N	Mean	SD	range	95% Confidence Interval for Mean
ICRS marcroscopic score	MSCs + HA trochlear	32	9.75	1.984	6–12	9.03–10.47
	HA trochlear	32	7.75	2.449	3–11	6.87–8.63
	control trochlear	32	5.91	2.176	2–9	5.12–6.69
	MSCs + HA condylar	32	8.84	2.216	3–11	8.04–9.64
	HA condylar	32	7	1.796	2–11	6.35–7.65
	control condylar	32	5.28	2.399	1–9	4.42–6.15
ICRS histological score	MSCs + HA trochlear	32	13.75	4.303	3–18	12.20–15.30
	HA trochlear	32	10.19	4.789	1–17	8.46–11.91
	control trochlear	32	6.44	3.388	3–15	5.22–7.66
	MSCs + HA condylar	32	12.53	4.189	3–18	11.02–14.04
	HA condylar	32	9.5	3.742	9–17	8.15–10.85
	control condylar	32	5.25	3.292	3–15	4.06–6.44

For the condylar defects, the ICRS macroscopic score for groups A, B and C were 8.84 ± 2.216, 7.00 ± 1.796, and 5.28 ± 2.399, respectively. Significant difference was found between groups A and B (*P = *0.001), groups B and C (P = 0.002) and groups A and C (*P* < 0.001) (Fig. 7c). Additionally, the ICRS histological score for groups A, B and C were 12.53 ± 4.189, 9.50 ± 3.742 and 5.25 ± 3.292 respectively. The score in group A was superior to those in group B (*P = *0.003) and group C (*P* < 0.001), and the score in group B was also superior to those in group C (*P* < 0.001) (Fig. 7d). These results suggested that both BMSCs plus HA and HA alone were effective in promoting the formation of neocartilage in cartilage defects and that adding BMSCs could improve the therapeutic effect.

## Discussion

In this study, we assessed the therapeutic effects of BMSCs and HA for cartilage defects in a beagle model. Our results showed that both BMSCs plus HA and HA alone could significantly promote neocartilage formation. However, BMSCs plus HA had a more prominent therapeutic effect on cartilage defects when compared to the HA alone. This therapeutic effect performed in the aspect of macroscopic analysis, MRI analysis, surface intact, osteochondral junction, matrix staining, neocartilage thickness and cell morphology.

Articular damage of cartilage defects is observed in trauma injury in people, especially athletes, and may cause pain and functional disability. The treatment of cartilage defects includes conventional treatments, physiotherapy, and surgical procedures and so on^25^. However, the abovementioned treatments can only delay the progression of articular cartilage damage but can not solve the problem fundamentally. Cartilage engineering can promote formation of neocartilage and has been commonly used to repair whole-layer cartilage defects in recent years^26^.

An ideal cartilage defect model is very important. In this study, 4-mm diameter acute (3 week) defects were induced successfully in canines that weighed 5–6 kg at 5–7 months old. This cartilage defect model was used in previous studies^27,28^. In Breinan’s study^27^, 4-mm diameter cartilage defects were successfully induced in 14 canines and used for further study. Many small animals (mice or rabbits) have been more popular and used for cartilage defect models in previous studies^15,29^. Canines are larger animals, and the biomechanics of canine’s knee joint is more similar to than are those of humans compared with mice and rabbits. And the beagles used in this study were moderately sized canine and represent a transition from a small-animal to a large-animal model. In the future, large-animal models could be used for further experimentation.

MSCs are a promising way to treat cartilage defects and have been used in many studies for knee articular damage with cartilage injury^9,10,30^. Autologous MSCs have been confirmed to provide excellent therapeutic effects in previous studies^31–33^. However, autologous cells are obtained from the patients themselves, which means that another invasive surgery is needed. Allogeneic BMSCs can be isolated from a variety of sources, are available for mass-production, and have a multipotent capability to produce cells via osteogenesis, adipogenesis and chondrogenesis^15^, which was also confirmed in our study. MSCs have been found to regenerate damaged cartilage, and inhibit fibrosis and inflammation without causing obvious rejection, because they can be safely transplanted^34,35^. The safety of immunomodulatory reactions of allogeneic MSCs *in vivo* also has been reported in Park’s study^35^. In some clinical studies^36–38^, MSCs have also been used without significant adverse reactions. A study by Park indicated that an allogeneic MSCs‐based novel medicinal product appeared to be safe and effective for regeneration of durable hyaline‐like cartilage over years of follow-up^39^. Another study by Gupta also found that intra-articular administration of allogeneic MSCs was safe^40^. Many studies also have found that BMSCs could promote cartilage repair and inhibit cartilage damage progression through a trophic mechanism via secreting cytokines^14,30^. In the present study, neocartilage-like cells were also observed in the defect sites 28 weeks after operation.

The quantity and quality of HA in synovial fluid are changed in dogs with OA and HA may be associated with early pathological changes in cartilage damage^41^. Intra-articular HA injection for the treatment of cartilage damage has been commonly used, but the efficacy is variable. Armstrong *et al*., observed that the use of intra-articular HA appeared to suppress progression of damage to cartilage and subchondral bone in early OA^42^. Clinical variables such as patient global assessment (PGA), walking pain (WP), and Western Ontario and McMaster Universities Osteoarthritis Index (WOMAC) decreased significantly after the injection of HA in a study conducted by Conrozier *et al*.^43^. A systematic review comparing corticosteroids with HA in knee articular disease showed that better efficacy was achieved with HA than with corticosteroids over the long term^44^. However, other studies hold opposite results. Altman *et al*. found no significant difference between HA and placebo in treating cartilage damage^45^. Other studies reported that HA did not change the progression of osteophytosis or fibrosis and did not improve the progression in knee articular disease^46–49^. This discrepancy might be because of improper injection position^50^. In addition, whether the improper or sub-optimal dosage of HA or prolonged interval between induction of cartilage damage and injection of HA could also affect the therapeutics need more evidence to confirm.

We took advantage of HA to promote cell migration^19^ and of BMSCs for their chondral differentiation.We found that BMSCs plus HA improved the therapeutic effect for cartilage defect in this study. An improvement in patients treated with allogeneic MSCs compared with HA was confirmed in a study by Vega *et al*.^30^. Lamo-Espinosa *et al*. found that BMSCs combined with HA was a safe and available treatment for improvement of knee articular disease in patients^51^. In animal models of articular cartilage damage, the efficacy of MSCs combined with HA has been found to be superior to those of MSCs or HA alone^15,52,53^. In the present study, we found that the macroscopic evaluation scores and histological assessment scale for BMSCs and HA were higher than those for HA alone with statistical significance for both femoral trochlear and condyle defects. Moreover, we also found that the macroscopic and histological scores for trochlear defects were a little higher than those for condylar defects when the same injection treatment was used but no statistical significance existed. The reason for this difference could be that the two defect parts were different on force, and further experiments are required to resolve this discrepancy. Additionally, it was unclear whether the cells improved cartilage repairing directly or whether improvement was caused by nutrition.

There were some limitation in this study that should be considered. First, our experiments were limited to the levels of cells and proteins and lacked of genetic and molecular exploration. Second, the repair of cartilage defects was a dynamic process, and our study was limited to the terminal point of repair. Third, a complex environment existed *in vivo*, and the rationale of MSCs and HA on cartilage repair was not explored. Fourth, this was a preliminary and non-blinded study, which could affect the evaluation of ICRS macroscopic and histological score. Further blinded and basic experiments are needed to improve understanding.

In summary, this study demonstrated that BMSCs plus HA could be a better way to repair cartilage defect in a beagle model. HA alone also contributed to improvement. This finding provides an available approach for the treatment of articular cartilage damage of cartilage defects. Further studies should be conducted that address the study limitation to confirm our findings.

## Materials and Methods

### Animal

This study was approved by the Institutional Animal Care and Use Committee of West China Hospital, Sichuan University, China. Twenty-seven male or female, weighed 5–6 kg at 5–7 months old beagles from the laboratory of Sichuan University were used in this study. All experimental procedures were performed following the guidelines of the care and use of laboratory animals.

### Mesenchymal stem cell isolation and cultivation

A total of 20 mL bone marrow aspirate mixtures were obtained from 3 male canines (24 canines left for further operation) prior to the transplantation procedure from the ilium. Allogeneic BMSCs were purified by density gradient centrifugation and centrifuged at 2500 rpm for 30 minutes. The cells were washed twice with phosphate-buffered saline (PBS) and resuspended in 10 mL of growth medium a-MEM (10% fetal bovine serum (FBS), 100 mg/mL streptomycin, 100 U/mL penicillin, 2 mmol/L l-glutamine and 25 ng/mL amphotericin B). BMSCs were incubated at 37 °C in a humidified incubator with 5% CO_2_. After 4 days, the culture medium was renewed, the nonadherent cells were removed, and the selected BMSCs were continued in the incubation. The medium was replaced twice a week. After the cultures had reached 70%–80% confluence, they were trypsinized with 0.25% trypsin/0.1% ethylene diamine tetraacetic acid (EDTA) for 3 minutes and passaged. Third-passage cultures (P3) were used in subsequent experiments. Flow cytometry was used to identify cell surface antigen. The P3 BMSCs were digested with 0.25% trypsin/0.1% EDTA and then were collected to give a cell suspension with 2 × 10^5^ cells/mL that was added to different EP tubes. CD29, CD34, CD44, CD45, CD90, CD105 and CD166 were tested by FACS Vantage Flow Cytometry (Beckman, USA). In addition, P3 MSCs cultured to 80% confluence were treated with appropriate media to confirm the multipotential differentiation of osteogenesis and chondrogenesis.

### Cartilage defect model and intervention *in vivo*

**Figure 8:**
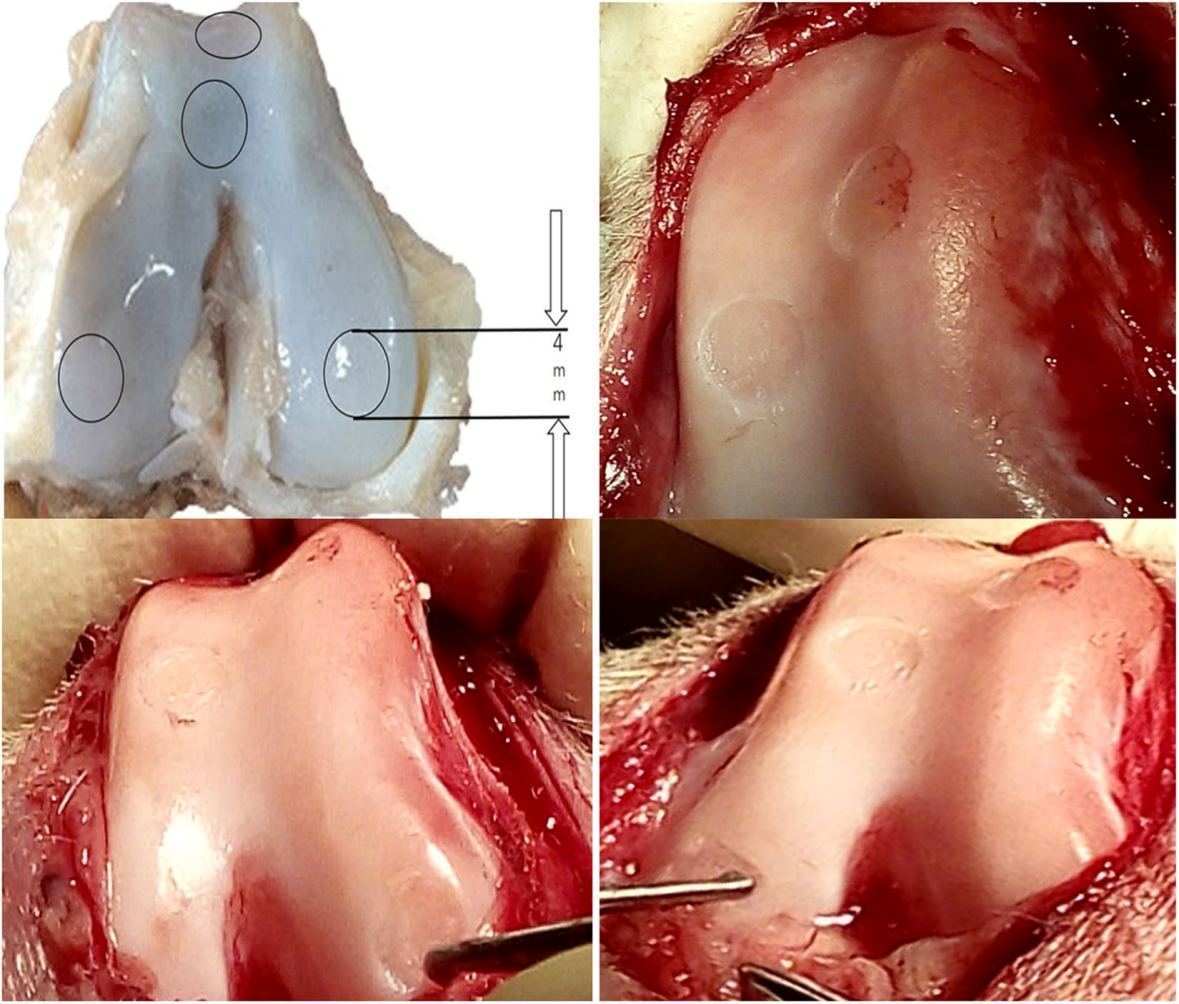
Operation of cartilage defect model in knee-joint.

Twenty-four healthy adult canines, male or female with weight ranging from 5.0 to 6.0 kg were used in this study. The canines were forbidden to eat or drink for 6 hours before operation. The canines received anesthesia via intraperitoneal injection with 3% sodium pentobarbital at a dosage of 1 mL/kg. After general disinfection of the knee, we made an incision on the medial knee joint, opened the joint capsule, dislocated the patella laterally and then exposed femoral condyles. Four cartilage defects (1 each for the medial and lateral condyle of the femur, and two for the trochlear groove of the femur) on each canine were created. A corneal trephine with a diameter of 4 mm was used to outline the cartilage defect site. Non-calcified cartilages were scraped away under loupe visualization, but calcified cartilage was not damaged (no bleeding from subchondral bone). The objective of modeling was to remove cartilage as much as possible without damaging the subchondral bone (Fig. 8). All canines were returned to separate cages after the operations and allowed to move freely. The animals received an antibiotic (800000 U/day penicillin for 3 days) and analgesic (0.12 mg/kg/day buprenorphine for 2 days) after operation. The canines were monitored for signs of activity, movement of joint, local infection, and other complications.

Three weeks after the operation, the 24 canines were randomly divided into three groups by injecting different therapeutic substances. We chose the lower and lateral edge of the patella to inject substances and sucked joint fluid back to confirm accurate puncture point. The three groups were as follows: (i) group A (BMSCs plus HA group, n = 8), had 1 × 10^7^ cells and 2 mL 1% HA (pH 6.7, 1000 kDa; Furuida, China) injected into the knee-joints cavity; (ii) group B (HA group, n = 8), had only 2 mL 1% HA injected into knee-joints; (iii) group C (control group with normal saline injected, n = 8).

### Gross appearance

Twenty-eight weeks after injection, the 24 canines (48 knees) were sacrificed. Surrounding soft tissues were removed and specimens of the defective cartilage were obtained. MRI was conducted to characterise the basic shape or cartilage-like signal at the defect sites. In addition, gross appearance, including the degree of defect repair, integration to border zone, and macroscopic appearance on the surface, were analyzed by two investigators. ICRS macroscopic evaluation of cartilage repair^54^ was also used to assess the treatment effect on condylar and trochlear defects. The ICRS macroscopic score was depicted as follows: 12 points indicated normal cartilage, and 0 points indicated severe damage. The higher the score, the better the cartilage repair (Table 1).

### Histological analysis

After all samples were washed twice with PBS (pH 7.4, Sigma), they were fixed in 4.0% paraformaldehyde for 7 days at 25–30 °C. And then they were decalcified in 10% formic acid for 3 months. After decalcification, the femoral condyles were cut into three pieces from the lateral to medial condyle along the sagittal plane. All samples were embedded in paraffin and cut into 5-μm sections. Prepared sections were stained with HE staining, Masson staining, and toluidine blue staining. The cell morphology, matrix color, surface intactness, cartilage thickness and integration with adjacent host cartilage were observed.

### Immunohistochemical analysis

The paraffin-embedded tissues were dewaxed by xylene. Type II collagen was retrieved with 1 mg/mL of pepsin (Sigma) in 0.5 M acetic acid at 37 °C for 30 minutes. Endogenous peroxidase was blocked with 3% hydrogen peroxide. The sections were rinsed with PBS and blocked with goat serum (Sichuan University Ltd, China). The sections were incubated with primary antibodies against type II collagen (Anti-Collagen II antibodies, ab34712, Abcam Trading, Shanghai, China) at 4 °C for 12 hours. The secondary antibodies (Peroxidase Affinipure Goat Anti-Mouse IgG (H + L), 115-035-003, Jackson, USA) were incubated at room temperature for 30 minutes as well as peroxidase–conjugated streptavidin (Sichuan University Ltd, China). And then the sections were washed with PBS for 5 minutes three times. Finally, a reagent of 3,3-diaminobenzidine solution containing 0.01% hydrogen peroxide was added and counterstaining was completed with hematoxylin.

### Histological score

To quantify the differences in histological and immunohistochemical staining, the ICRS Visual Histological Assessment score was evaluated according to the method of Varlet *et al*.^55^. In brief, a scale from 18 (good) to 0 (severe) was used to assess condylar and trochlear defects. Surface, matrix, cell distribution, cell population viability, subchondral bone, and cartilage mineralization (calcified cartilage) were assessed (Table 2).

### Statistical analysis

The SPSS 22.0 software (SPSS Inc, Chicago, USA) was used for the statistical analyses. All data are reported as means ± SD. Student-Newman-Keuls was performed to compare effects on new cartilage formation among groups. A *P* value < 0.05 was considered significant.

### Data availability

No datasets were generated or analyzed during the current study and all data come from the listed authors.

## Electronic supplementary material

Supplementary Information

## References

[1] Hunziker EB (1999). Articular cartilage repair: are the intrinsic biological constraints undermining this process insuperable?. Osteoarthritis and cartilage/OARS, Osteoarthritis Research Society.

[2] Hong E, Reddi AH (2012). MicroRNAs in chondrogenesis, articular cartilage, and osteoarthritis: implications for tissue engineering. Tissue engineering. Part B, Reviews.

[3] Jevsevar, D. S. Treatment of osteoarthritis of the knee: evidence-based guideline, 2nd edition. *The Journal of the American Academy of Orthopaedic Surgeons* **21**, 571-576, 10.5435/JAAOS-21-09-571 (2013). 10.5435/JAAOS-21-09-571 23996988

[4] Wang HM, Liu JN, Zhao Y (2010). Progress on integrated Chinese and Western medicine in the treatment of osteoarthritis. Chinese journal of integrative medicine.

[5] Simon LS (1999). Osteoarthritis. Current rheumatology reports.

[6] Reddi AH (1998). Role of morphogenetic proteins in skeletal tissue engineering and regeneration. Nature biotechnology.

[7] Beris AE, Lykissas MG, Papageorgiou CD, Georgoulis AD (2005). Advances in articular cartilage repair. Injury.

[8] Kim YS (2015). Comparative Matched-Pair Analysis of the Injection Versus Implantation of Mesenchymal Stem Cells for Knee Osteoarthritis. The American journal of sports medicine.

[9] Yang X (2015). Intraarticular Injection of Allogenic Mesenchymal Stem Cells has a Protective Role for the Osteoarthritis. Chinese medical journal.

[10] Toghraie FS (2011). Treatment of osteoarthritis with infrapatellar fat pad derived mesenchymal stem cells in Rabbit. The Knee.

[11] Cuervo B (2014). Hip osteoarthritis in dogs: a randomized study using mesenchymal stem cells from adipose tissue and plasma rich in growth factors. International journal of molecular sciences.

[12] Emadedin, M. *et al*. Long-Term Follow-up of Intra-articular Injection of Autologous Mesenchymal Stem Cells in Patients with Knee, Ankle, or Hip Osteoarthritis. *Archives of Iranian medicine* **18**, 336–344, doi:015186/AIM.003 (2015). 26058927

[13] Koh YG, Choi YJ (2012). Infrapatellar fat pad-derived mesenchymal stem cell therapy for knee osteoarthritis. The Knee.

[14] Cui YP (2015). Bone marrow mesenchymal stem cells in Sprague-Dawley rat model of osteoarthritis. Beijing da xue xue bao. Yi xue ban=Journal of Peking University. Health sciences.

[15] Chiang ER (2016). Allogeneic Mesenchymal Stem Cells in Combination with Hyaluronic Acid for the Treatment of Osteoarthritis in Rabbits. PloS one.

[16] Watterson JR, Esdaile JM (2000). Viscosupplementation: therapeutic mechanisms and clinical potential in osteoarthritis of the knee. The Journal of the American Academy of Orthopaedic Surgeons.

[17] Moreland LW (2003). Intra-articular hyaluronan (hyaluronic acid) and hylans for the treatment of osteoarthritis: mechanisms of action. Arthritis research & therapy.

[18] Clegg TE, Caborn D, Mauffrey C (2013). Viscosupplementation with hyaluronic acid in the treatment for cartilage lesions: a review of current evidence and future directions. European journal of orthopaedic surgery & traumatology: orthopedie traumatologie.

[19] Murashita T, Nakayama Y, Hirano T, Ohashi S (1996). Acceleration of granulation tissue ingrowth by hyaluronic acid in artificial skin. British journal of plastic surgery.

[20] Ehlers EM, Behrens P, Wunsch L, Kuhnel W, Russlies M (2001). Effects of hyaluronic acid on the morphology and proliferation of human chondrocytes in primary cell culture. Annals of anatomy=Anatomischer Anzeiger: official organ of the Anatomische Gesellschaft.

[21] Askari A (2016). Hyaluronic acid compared with corticosteroid injections for the treatment of osteoarthritis of the knee: a randomized control trail. SpringerPlus.

[22] Auerbach B, Melzer C (2002). Cross-linked hyaluronic acid in the treatment of osteoarthritis of the knee–results of a prospective randomized trial. Zentralblatt fur Chirurgie.

[23] Corvelli M, Che B, Saeui C, Singh A, Elisseeff J (2015). Biodynamic performance of hyaluronic acid versus synovial fluid of the knee in osteoarthritis. Methods.

[24] Ghosh P, Holbert C, Read R, Armstrong S (1995). Hyaluronic acid (hyaluronan) in experimental osteoarthritis. The Journal of rheumatology. Supplement.

[25] Manjhi J, Gupta M, Sinha A, Rawat B, Rai DV (2016). Effects of Balsamodendron mukul Gum Resin Extract on Articular Cartilage in Papain-induced Osteoarthritis. Alternative therapies in health and medicine.

[26] Vinatier C, Guicheux J (2016). Cartilage tissue engineering: From biomaterials and stem cells to osteoarthritis treatments. Annals of physical and rehabilitation medicine.

[27] Breinan HA (1997). Effect of cultured autologous chondrocytes on repair of chondral defects in a canine model. The Journal of bone and joint surgery. American volume.

[28] Shortkroff S (1996). Healing of chondral and osteochondral defects in a canine model: the role of cultured chondrocytes in regeneration of articular cartilage. Biomaterials.

[29] Mak J (2016). Intra-articular injection of synovial mesenchymal stem cells improves cartilage repair in a mouse injury model. Scientific reports.

[30] Vega A (2015). Treatment of Knee Osteoarthritis With Allogeneic Bone Marrow Mesenchymal Stem Cells: A Randomized Controlled Trial. Transplantation.

[31] Caminal M (2014). Use of a chronic model of articular cartilage and meniscal injury for the assessment of long-term effects after autologous mesenchymal stromal cell treatment in sheep. New biotechnology.

[32] Wong KL (2013). Injectable cultured bone marrow-derived mesenchymal stem cells in varus knees with cartilage defects undergoing high tibial osteotomy: a prospective, randomized controlled clinical trial with 2 years’ follow-up. Arthroscopy: the journal of arthroscopic & related surgery: official publication of the Arthroscopy Association of North America and the International Arthroscopy Association.

[33] Orozco L (2014). Treatment of knee osteoarthritis with autologous mesenchymal stem cells: two-year follow-up results. Transplantation.

[34] Voswinkel J, Chapel A (2012). Mesenchymal stem cells and rheumatism. State of the art. Zeitschrift fur Rheumatologie.

[35] Park SA (2013). Safety and immunomodulatory effects of allogeneic canine adipose-derived mesenchymal stromal cells transplanted into the region of the lacrimal gland, the gland of the third eyelid and the knee joint. Cytotherapy.

[36] Kim YS (2015). Mesenchymal stem cell implantation in osteoarthritic knees: is fibrin glue effective as a scaffold?. The American journal of sports medicine.

[37] Jo CH (2014). Intra-articular injection of mesenchymal stem cells for the treatment of osteoarthritis of the knee: a proof-of-concept clinical trial. Stem cells.

[38] Wang Y (2017). Safety, tolerability, clinical, and joint structural outcomes of a single intra-articular injection of allogeneic mesenchymal precursor cells in patients following anterior cruciate ligament reconstruction: a controlled double-blind randomised trial. Arthritis research & therapy.

[39] Park YB, Ha CW, Lee CH, Yoon YC, Park YG (2017). Cartilage Regeneration in Osteoarthritic Patients by a Composite of Allogeneic Umbilical Cord Blood-Derived Mesenchymal Stem Cells and Hyaluronate Hydrogel: Results from a Clinical Trial for Safety and Proof-of-Concept with 7 Years of Extended Follow-Up. Stem cells translational medicine.

[40] Gupta PK (2016). Efficacy and safety of adult human bone marrow-derived, cultured, pooled, allogeneic mesenchymal stromal cells (Stempeucel(R)): preclinical and clinical trial in osteoarthritis of the knee joint. Arthritis research & therapy.

[41] Venable RO, Stoker AM, Cook CR, Cockrell MK, Cook JL (2008). Examination of synovial fluid hyaluronan quantity and quality in stifle joints of dogs with osteoarthritis. American journal of veterinary research.

[42] Armstrong S, Read R, Ghosh P (1994). The effects of intraarticular hyaluronan on cartilage and subchondral bone changes in an ovine model of early osteoarthritis. The Journal of rheumatology.

[43] Conrozier T (2009). Safety, efficacy and predictive factors of efficacy of a single intra-articular injection of non-animal-stabilized-hyaluronic-acid in the hip joint: results of a standardized follow-up of patients treated for hip osteoarthritis in daily practice. Archives of orthopaedic and trauma surgery.

[44] Iannitti T, Lodi D, Palmieri B (2011). Intra-articular injections for the treatment of osteoarthritis: focus on the clinical use of hyaluronic acid. Drugs in R&D.

[45] Altman RD, Akermark C, Beaulieu AD, Schnitzer T (2004). & Durolane International Study, G. Efficacy and safety of a single intra-articular injection of non-animal stabilized hyaluronic acid (NASHA) in patients with osteoarthritis of the knee. Osteoarthritis and cartilage/OARS, Osteoarthritis Research Society.

[46] Smith GN, Myers SL, Brandt KD, Mickler EA (1998). Effect of intraarticular hyaluronan injection in experimental canine osteoarthritis. Arthritis and rheumatism.

[47] Smith G (2005). Effect of intraarticular hyaluronan injection on vertical ground reaction force and progression of osteoarthritis after anterior cruciate ligament transection. The Journal of rheumatology.

[48] Munteanu SE (2011). Effectiveness of intra-articular hyaluronan (Synvisc, hylan G-F 20) for the treatment of first metatarsophalangeal joint osteoarthritis: a randomised placebo-controlled trial. Annals of the rheumatic diseases.

[49] Jorgensen A (2010). Intra-articular hyaluronan is without clinical effect in knee osteoarthritis: a multicentre, randomised, placebo-controlled, double-blind study of 337 patients followed for 1 year. Annals of the rheumatic diseases.

[50] Abate M, Schiavone C, Salini V (2012). Hyaluronic acid in ankle osteoarthritis: why evidence of efficacy is still lacking?. Clinical and experimental rheumatology.

[51] Lamo-Espinosa JM (2016). Intra-articular injection of two different doses of autologous bone marrow mesenchymal stem cells versus hyaluronic acid in the treatment of knee osteoarthritis: multicenter randomized controlled clinical trial (phase I/II). Journal of translational medicine.

[52] Suhaeb AM, Naveen S, Mansor A, Kamarul T (2012). Hyaluronic acid with or without bone marrow derived-mesenchymal stem cells improves osteoarthritic knee changes in rat model: a preliminary report. Indian journal of experimental biology.

[53] Sato M (2012). Direct transplantation of mesenchymal stem cells into the knee joints of Hartley strain guinea pigs with spontaneous osteoarthritis. Arthritis research & therapy.

[54] van den Borne MP (2007). International Cartilage Repair Society (ICRS) and Oswestry macroscopic cartilage evaluation scores validated for use in Autologous Chondrocyte Implantation (ACI) and microfracture. Osteoarthritis and cartilage/OARS, Osteoarthritis Research Society.

[55] Varlet PM (2003). Histological assessment of Cartilage Repair. The journal of bone and joint surgery.

