# Supplementary material for: Mesenchymal Stem Cells in Combination with Hyaluronic Acid for Articular Cartilage Defects
Source: Sci Rep. 2018 Jul 2;8:9900. doi: 10.1038/s41598-018-27737-y (PMC6028658; doi:10.1038/s41598-018-27737-y)
Supplement: Supplementary file 1 — Supplementary Information [file 41598_2018_27737_MOESM1_ESM.pdf]

Title Page

Mesenchymal Stem Cells in Combination with Hyaluronic Acid for Articular Cartilage Defects

Lang Li[1*], Xin Duan[1*], Zhaoxin Fan[2*], Long Chen[1,3], Fei Xing[1], Zhao Xu[4], Qiang Chen[2,5], Zhou Xiang[1**]

[1]Department of Orthopedics, West China Hospital, Sichuan University, Chengdu, Sichuan, China, Post code: 610016
[2]Sichuan Cord Blood Bank, Jinquanlu15#, Chengdu, Sichuan, China, Post code: 610016
[3]Department of Orthopedics, Guizhou Provincial People's Hospital, Guiyang, Guizhou, China, Post code: 550002
[4]Department of Anesthesia, West China Hospital, Sichuan University, Chengdu, Sichuan, China, Post code: 610016
[5]Institude of Blood Transfusion, Chinese Academy of Medical Science, Sichuan, China, Post code: 610016

[**]Corresponding author: Zhou Xiang, Orthopedics Department, West China Hospital, Sichuan University, #37 Guoxuexiang, Chengdu, Sichuan province, China. Post code: 610016
Phone-Number: +86 28 85423426, Fax-Number: +86 28 85423438, E-mail: xiangzhou15@hotmail.com

[*]These authors contributed equally to this work.

Email：
Email For:

Lang Li: 578664804@qq.com
Xin Duan: 45861793@qq.com
Zhaoxin Fan: 3451491841@qq.com
Long Chen: 776377010@qq.com
Fei Xing: 408451184@qq.com
Zhao Xu: 86325607@qq.com
Qiang Chen: 1424545388@qq.com

Supplementary Information

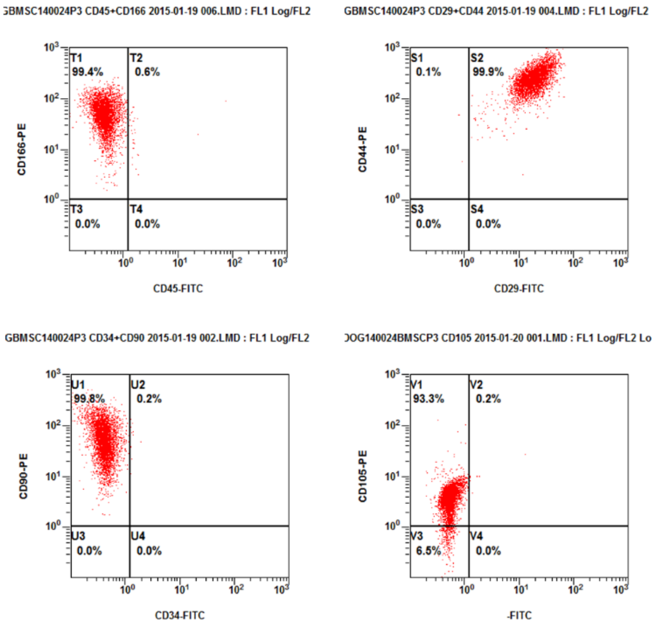

Fig S1

S1 File (Figure S1). Immunophenotyping analyses of BMSC.

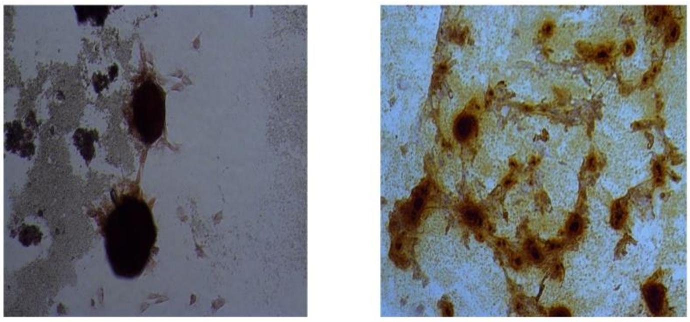

A Fig S2 B

S2 File (Figure S2). Osteogenesis and chondrogenesis of BMSCs in vitro. (A) Chondrogenesis differentiation of BMSC and Alizarin red staining ;(B) Osteogenesis differentiation of BMSC and Safarin O staining.
